# New Triterpenoids with Cytotoxic Activity from *Actinidia Valvata*

**DOI:** 10.3390/ijms131114865

**Published:** 2012-11-13

**Authors:** Li-Ping Qu, Guo-Yin Zheng, Yong-Hua Su, Hui-Qing Zhang, Yan-Long Yang, Hai-Liang Xin, Chang-Quan Ling

**Affiliations:** Department of Traditional Chinese Medicine, Changhai Hospital of Second Military Medical University, Shanghai 200433, China; E-Mails: doudou0586@yahoo.com.cn (L.-P.Q.); herbzheng@163.com (G.-Y.Z.); suyh2001@126.com (Y.-H.S.); newdew628@yahoo.com.cn (H.-Q.Z.); yanlongyangzy@163.com (Y.-L.Y.)

**Keywords:** *Actinidia valvata* Dunn, triterpenoid, cytotoxic activity

## Abstract

Two new triterpenoids, 30-*O*-β-d-glucopyranosyloxy-2α,3α,24-trihydroxyurs-12, 18-diene-28-oic acid *O*-β-d-glucopyranosyl ester (**1**) and 2α,3β,3,30-tetrahydroxyurs-12, 18-diene-28-oic acid *O*-β-d-glucopyranosyl ester (**2**) were isolated from roots of *Actinidia valvata* Dunn. Their structures were elucidated by means of extensive spectroscopic studies. Both these two new compounds showed moderate cytotoxic activity *in vitro* against BEL-7402 and SMMC-7721 tumor cell line.

## 1. Introduction

*Actinidia valvata* Dunn is a shrub mainly growing in eastern China (Zhejiang and Jiangxi province) [1]. Its roots known as “mao ren shen” in traditional Chinese medicine exhibit anti-tumor and anti-inflammatory activity, and have been used for many years in the treatment of hepatoma, lung carcinoma and myeloma [2,3]. As we all know, hepatoma is very difficult to treat, and active components from medicinal herbs may be effective for research and development of new drugs [4]. In a previous study, we have carried out screening for cytotoxic activity of “mao ren shen”, and two new polyoxygenated triterpenoids, 2β,3α,6α,20α,24,30-hexahydroxyurs-12-en-28-oic acid and 2β,3α,20β,23,24,30-hexahydroxyurs-12-en-28-oic acid *O*-β-d-glucopyranosyl ester were separated [5]. In this paper, two new triterpenoid saponins with cytotoxic activity against BEL-7402 and SMMC-7721 tumor cell line are reported.

## 2. Results and Discussion

**Figure 1:**
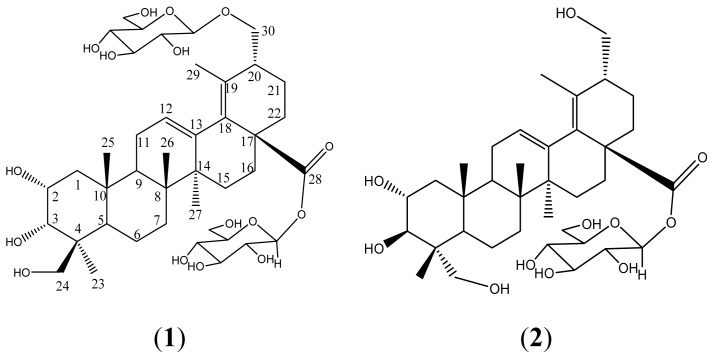
Structures of compound **1** and **2**.

The roots of *Actinidia valvata* Dunn were extracted with 80% EtOH. The concentrated extract was suspended in H_2_O and successively extracted with petroleum ether (60°–90°), AcOEt, and *n*-BuOH. The *n*-BuOH-soluble extract was repeatedly subjected to column chromatography to yield compound **1**,**2**Figure 1. Both these compounds were triterpenoid saponins with 12,18-diene-urs skeleton. Their structures were elucidated by detailed spectroscopic analysis.

Compound **1** was a white amorphous powder, displayed positive *Liebermann*–*Burchard* test, was optically active with [α]_D_^25^ = 5.89 (*c* = 0.1, MeOH), and had the molecular formula C_42_H_66_O_16_, with ten degrees of unsaturation, as determined according to a *pseudo*-molecular-ion peaks at 849.4240 ([*M* + Na]^+^; calc. 849.4249) in the HR-ESI-MS.

^13^C-NMR (DEPT) spectra of compound **1** revealed 42 carbon signals, including five CH_3_, two C=C bonds (tri-, four-substituted) and one C=O group. Assuming compound **1** has skeleton of urs-triterpenoid, the assignment of the two C=C bonds should be highly concerned. In ^1^H-NMR spectra of compound **1**, five singlets at δ(H) 0.80–1.89 consisting of five CH_3_, and proton signal at δ(H) 5.53 (br) was assigned to 12-position. In HMBC spectra, clear correlation of 19–CH_3_ with two quaternary *C*-atoms at δ(C) 126.17 (*s*) and δ(C) 136.92 (*s*) was observed, and the correlation signals of 12-H with these two quaternary *C*-atoms were also observed. Thus, the two C=C bonds can be rightly assigned to 18-, 19-position, respectively. The HMBC correlation signal of 20-H with *C*-atom at δ(C) 69.68(t), was observed, then the oxygenation of 30-C may be deduced successfully. In NOSEY spectra, as the correlations of 2-H with 3-H, 2-H with 10-Me, 8-Me with 10-Me were observed, and by comparing with reference data [6,7], the configuration of 2α-OH, 3α-OH, and the 24-OH can be confirmed. ^1^H-NMR and ^13^C-NMR data of glycon moiety of compound **1** indicated it featured two glucopyranosyls. The *C*-atom at δ(C) 95.82(d) and H (5.39, d, *J* = 8.0 Hz), *C*-atom at δ(C) 101.85(d) and H (4.20, d, *J* = 8.0 Hz) were assigned as anomeric *C*-atoms and prontons, respectively. The linkage of two glucopyranosyls with aglycone maybe deduced by correlation of anomeric pronton with glycosidated *C*-atoms in HMBC spectra [H (5.39, d, *J* = 8.0 Hz) to C=O, H (4.20, d, *J* = 8.0 Hz) to *C*-atom at 69.68(t)].

**Figure 2:**
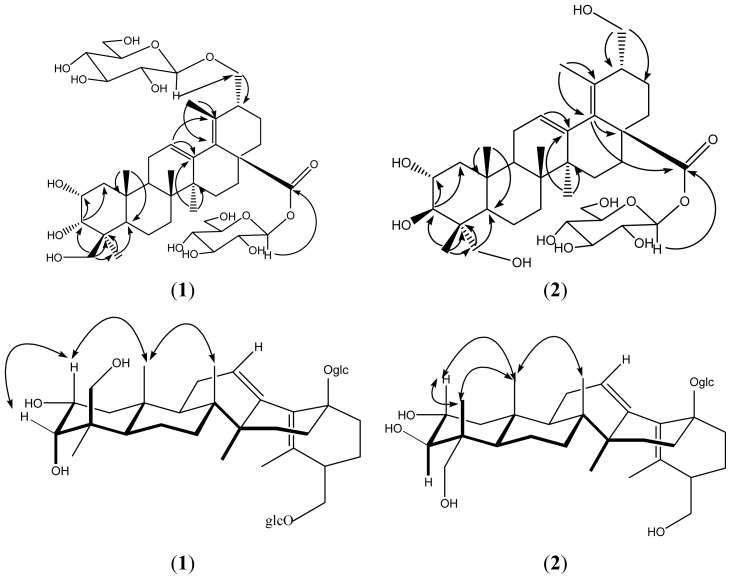
Key HMBC (→) and NOESY (↔) correlations of compound **1** and **2**.

Basing on above analysis, in combination with the ^1^H-, ^13^C-NMR spectra (Table 1), HMQC, HMBC, and NOSEY data (Figure 2), established the structure of compound **1** as 30-*O*-β-d-glucopyranosyloxy- 2α,3α,24-trihydroxyurs-12,18-diene-28-oic acid *O*-β-d-glucopyranosyl ester (**1**).

The compound **2** was obtained as white amorphous powder, displayed positive *Liebermann*–*Burchard* test, was optically active with [α]_D_^25^ = 7.35 (*c* = 0.1, MeOH), and had the molecular formula C_36_H_56_O_11_ with nine degrees of unsaturation, as determined according to a *pseudo*-molecular-ion peaks at 687.3717 ([*M* + Na]^+^; calc. 687.3720) in the HR-ESI-MS.

Comparing the ^1^H-NMR and ^13^C-NMR (Table 1) spectra, compound **2** has only a 28-glucopyranosyl. The configuration of 2α-OH, 3β-OH, and 23-OH can be confirmed by analyzing the correlations of NOSEY spectra and comparing with reference data [6,7]. In combination with the ^1^H-, ^13^C-NMR spectra (Table 1), HMQC, HMBC, and NOSEY data (Figure 2), established the structure of compound **2** as 2α,3β,23,30-tetrahydroxyurs-12, 18-diene-28-oic acid *O*-β-d-glucopyranosyl ester (**2**).

Compounds **1** and **2** showed moderate *in vitro* cytotoxic activity against BEL-7402 (IC_50_ value of 92.2 and 89.7 μg/mL, resp.) and SMMC-7721 (IC_50_ value of 58.1 and 89.7 μg/mL, resp.), as determined by classical MTT (3-(4,5-dimethylthiazol-2-yl)-2,5-diphenyl-2*H*-tetrazolium bromide) colorimetric assay.

## 3. Experimental Section

### 3.1. General

Silica-gel plates (Sinopharm Chemical Reagent Co., Ltd.) were used for TLC analysis. mp: WRS-1A micro-melting-point apparatus; uncorrected. Optical rotations: JASCO P-1300 spectropolarimeter. IR: Spectra: BRUKER VECTOR-22 spectrophotometer; in cm^−1. 1^H-, ^13^C-, 2D-NMR spectra: BRUKER AVANCE 600 spectrometer; chemical shifts δ in ppm rel. to (CH_3_)_4_Si, coupling constant *J* in Hz. ESI-MS: Finnigan LCQ mass spectrometer; in m/z. HR-ESI-MS: Q-Tof micro YA019 mass spectrometer.

### 3.2. Material

The roots of *Actinidia valvata* Dunn were collected in Changshan County, Zhejiang Province, China, in October 2006, and identified by Zheng Han-Chen, Department of pharmacognosy, School of pharmacy, Second military medical university. A voucher specimen (No. 20061005) was deposited at Department of pharmacognosy, School of pharmacy, Second military medical university.

### 3.3. Extraction and Isolation

The powdered plant material of roots of *Actinidia valvata* Dunn 30 kg was refluxed with 8 times of 80% EtOH solution for 3 times, 1.5 h each time. The extract was concentrated under reduced pressure to brown syrup, which was partitioned between H_2_O and petroleum ether (PE), AcOEt, and BuOH, successively. The *n*-BuOH soluble fraction (280.6 g) was subjected to column chromatography (CC) on silica gel (SiO_2_), eluting with CHCl_3_/MeOH/H_2_O (10:1:0.1 to 2:1 0.1) to afford 9 fraction 1–9. Fraction 5 were repeatedly subjected to CC (Pharmadex LH-20 and RP C-18) to yield compound **1** (10.4 mg) and 2 (14.6 mg).

30-*O*-β-d-glucopyranosyloxy-2α,3α,24-trihydroxyurs-12, 18-diene-28-oic acid *O*-β-d-glucopyranosyl ester (**1**): white amorphous powder, mp 140°–142°, [α]_D_^25^ = 5.89 (*c* = 0.1, MeOH). IR (KBr): 3432, 2920, 2852, 1641, 1380, 1038. ^1^H-NMR (600 MHz, C_5_D_5_N) and ^13^C-NMR (150 MHz, C_5_D_5_N): Table 1. ESI-MS: 849.48 ([*M* + Na]^+^), HR-ESI-MS: 849.4240 ([*M* + Na]^+^, C_42_H_66_N_a_O_16_^+^, calc. 849.4249).

2α,3β,23,30-tetrahydroxyurs-12, 18-diene-28-oic acid *O*-β-d-glucopyranosyl ester (**2**): white amorphous powder. mp 220° (carbonification), [α]_D_^25^ = 7.35 (*c* = 0.1, MeOH). IR (KBr): 3448, 2963, 1681, 1644, 1381, 1278, 1080. ^1^H-NMR (600 MHz, C_5_D_5_N) and ^13^C-NMR (150 MHz, C_5_D_5_N): Table 1 ESI-MS: 687.89 ([*M* + Na]^+^), HR-ESI-MS: 687.3717 ([*M* + Na]^+^, C_36_H_56_N_a_O_11_^+^, calc. 687.3720).

## 4. Conclusions

In the present research, two new triterpenoids, 30-*O*-β-d-glucopyranosyloxy-2α,3α,24-trihydroxyurs-12, 18-diene-28-oic acid *O*-β-d-glucopyranosyl ester and 2α,3β,23,30-tetrahydroxyurs-12, 18-diene-28-oic acid *O*-β-d-glucopyranosyl ester were isolated from roots of *Actinidia valvata* Dunn, and their structures were elucidated by means of extensive spectroscopic studies. Both these new compounds showed moderate cytotoxic activity *in vitro* against BEL-7402 and SMMC-7721 tumor cell line.

## Figures and Tables

**Table 1:** ^1^H and ^13^C-NMR Data of 1 and 2 (in C_5_D_5_N). δ in ppm, *J* in Hz.

Position	1		2	
	
	δ(C) a	δ(H) b	δ(C) a	δ(H) b
1	42.81 (t)	1.31 (dd, *J* = 12,4.2), 2.19 (dd, *J* = 12,4.2)	48.03 (*t*)	1.30 (*m*), 2.29 (*m*)
2	66.98 (d)	3.88 (*m*)	68.93 (*d*)	4.25 (*m*)
3	74.90 (d)	3.70 (d, *J* = 2.0)	78.18 (*d*)	4.19 (*m*)
4	45.36 (s)	—	43.61 (*s*)	—
5	49.94 (d)	1.79 (*m*)	48.31 (*d*)	1.73 (*m*)
6	19.15 (t)	1.33 (*m*)	18.41 (*t*)	1.04 (*m*), 1.15 (*m*)
7	33.23 (t)	1.28 (*m*), 1.30 (*m*)	33.86 (*t*)	1.31 (*m*), 1.75 (*m*)
8	40.67 (s)	—	39.80 (*s*)	—
9	48.96 (d)	1.71 (*m*)	48.03 (*d*)	1.83 (*m*)
10	39.07 (s)	—	38.31 (*s*)	—
11	24.25 (t)	1.74 (*m*), 1.93 (*m*)	23.64 (*t*)	1.74 (*m*), 2.01 (*m*)
12	129.19 (d)	5.53 (*br*)	127.98 (*d*)	5.60 (*br*)
13	137.97 (s)	—	137.53 (*s*)	—
14	44.69 (s)	—	43.90 (*s*)	—
15	29.09 (t)	1.09 (*m*), 1.88 (*m*)	28.58 (*t*)	1.06 (*m*), 2.25 (*m*)
16	24.72 (t)	1.74 (*m*), 1.98 (*m*)	32.97 (*t*)	1.79 (*m*), 1.98 (*m*)
17	48.83 (s)	—	47.25 (*s*)	—
18	126.17 (s)	—	129.59 (*s*)	—
19	136.92 (s)	—	131.27 (*s*)	—
20	51.45 (s)	3.30 (*br*)	50.74 (*d*)	3.55 (*br*)
21	24.67 (t)	1.97 (*m*)	23.87 (*t*)	1.99 (*m*)
22	35.19 (t)	1.28 (*m*), 1.53 (*m*)	24.41 (*t*)	2.06 (*m*)
23	22.56 (q)	1.07 (*s*)	66.52 (*t*)	3.66 (d, *J* = 10.2), 4.16 (d, *J* = 10.2)
24	65.72 (t)	3.36 (d, *J* = 8.0), 3.68 (d, *J* = 8.0)	14.42 (*q*)	1.03 (*s*)
25	17.86 (q)	0.94 (*s*)	17.95 (*q*)	1.08 (*s*)
26	18.21 (q)	0.85 (*s*)	18.27 (*q*)	1.15 (*s*)
27	23.17 (q)	1.89 (*s*)	22.23 (*q*)	0.97 (*s*)
28	178.07 (s)	—	176.24 (*s*)	—
29	17.44 (q)	1.68 (*s*)	16.83 (*q*)	1.75 (*s*)
30	69.68 (t)	4.09 (d, *J* = 20.0), 4.45 (d, *J* = 20.0)	62.20 (*t*)	4.38 (d, *J* = 10.2)
28-glc-1	95.82 (d)	5.39 (d, 8)	95.82 (*d*)	5.60 (d, *J* = 8.0)
2	73.75 (d)	3.31 (*m*)	74.19 (*d*)	3.31 (*m*)
3	77.91 (d)	3.33 (*m*)	78.84 (*d*)	3.33 (*m*)
4	71.46 (d)	3.33 (*m*)	71.12 (*d*)	3.33 (*m*)
5	78.54 (d)	3.38 (*m*)	79.25 (*d*)	3.38 (*m*)
6	62.49 (t)	3.64 (dd, *J* = 12.0,1.8), 3.77 (dd, *J* = 12.0, 1.8)	62.99 (*t*)	3.65 (dd, *J* = 12.0, 1.8), 3.78 (dd, *J* = 12.0, 1.8)
30-glc-1	101.85 (d)	4.20 (d, *J* = 8.0)		
2	74.53 (d)	3.31 (*m*)		
3	77.83 (d)	3.33 (*m*)		
4	70.97 (d)	3.33 (*m*)		
5	77.95 (d)	3.38 (*m*)		
6	62.30 (t)	3.63 (dd, *J* = 12.0, 1.8), 3.76 (dd, *J* = 12.0, 1.8)		

a Recorded at 150 MHz, multiplicity by DEPT;

b Recorded at 600 MHz.
